# IMPLEMENTING AND MEASURING IMPACT OF A FIVE-YEAR TRANSLATIONAL GERIATRIC ONCOLOGY RESEARCH PROGRAM

**DOI:** 10.1093/geroni/igac059.934

**Published:** 2022-12-20

**Authors:** Irene Blackberry, Christopher Steer, Tshepo Rasekaba, Stacey Rich, Nicole Webb, Kim Young

**Affiliations:** La Trobe University, Wodonga, Victoria, Australia; Border Medical Oncology and Haematology, Albury, New South Wales, Australia; La Trobe University, Wodonga, Victoria, Australia; La Trobe University, John Richards Centre, Wodonga, Victoria, Australia; Border Medical Oncology and Haematology, Albury, New South Wales, Australia; La Trobe University, Wodonga, Victoria, Australia

## Abstract

The Geriatric Oncology Regional Victorian Trials Alliance, Linkages, Special populations, Equity program (GO ReViTALISE) is part of a five-year state-wide grant to increase access to cancer clinical trials in regional Victoria, Australia. The initiative covers engagement with key stakeholders and trial sites, co-design of research programs with consumers, implementation of multiple interventional and non-interventional research, and capacity building. Employing a framework prospectively is critical to guide and scaffold implementation of complex interventions and capture the process of how several activities interact. Likewise, there is a need to show research impact beyond successful implementation and clinical outcomes. Using the GO ReViTALISE as a case study, we will demonstrate how an implementation framework and the Framework to Assess the Impact from Translational health research can deliver a cohesive, impactful and measurable program of research to increase older adults’ participation in cancer clinical trials, trials availability and build capacity in regional cancer sites.

